# Drug Delivery Innovations for Enhancing the Anticancer Potential of Vitamin E Isoforms and Their Derivatives

**DOI:** 10.1155/2015/584862

**Published:** 2015-06-07

**Authors:** Christiana M. Neophytou, Andreas I. Constantinou

**Affiliations:** Department of Biological Sciences, Faculty of Pure and Applied Sciences, University of Cyprus, 1678 Nicosia, Cyprus

## Abstract

Vitamin E isoforms have been extensively studied for their anticancer properties. Novel drug delivery systems (DDS) that include liposomes, nanoparticles, and micelles are actively being developed to improve Vitamin E delivery. Furthermore, several drug delivery systems that incorporate Vitamin E isoforms have been synthesized in order to increase the bioavailability of chemotherapeutic agents or to provide a synergistic effect. D-alpha-tocopheryl polyethylene glycol succinate (Vitamin E TPGS or TPGS) is a synthetic derivative of natural alpha-tocopherol which is gaining increasing interest in the development of drug delivery systems and has also shown promising anticancer effect as a single agent. This review provides a summary of the properties and anticancer effects of the most potent Vitamin E isoforms and an overview of the various formulations developed to improve their efficacy, with an emphasis on the use of TPGS in drug delivery approaches.

## 1. Introduction

Vitamin E exists in nature in 8 natural isoforms that have exhibited diverse therapeutic properties in numerous studies over the last decades.* In vitro*,* in vivo,* and clinical studies of the Vitamin E isoforms have highlighted their antioxidant, anti-inflammatory, neuroprotective, and antithrombotic abilities among others [1–3]. Importantly, Vitamin E natural isoforms have been shown to act in a preventive and therapeutic manner against several types of cancer and are still being widely investigated for their potential efficacy in this disease [4].

Even though* in vitro* studies have highlighted the great anticancer potential of Vitamin E isoforms, their anticancer efficacy in animal models is hindered due to low compound bioavailability and solubility. The* in vivo* absorption of Vitamin E isoforms is limited due to their lipophilic nature and insolubility in aqueous solvents [5, 6]. Their formulations in ethanol, DMSO, or vegetable oil emulsions limit their applications in the clinical setting. Furthermore, when Vitamin E isoforms are present in high concentration in the intestine, their absorption is reduced because their transport becomes saturated [7, 8]. As described in detail below, Vitamin E isoforms also display nonspecific distribution in tissues and when administered at high concentrations they are rapidly metabolized and excreted in urine.

**Figure 1:**
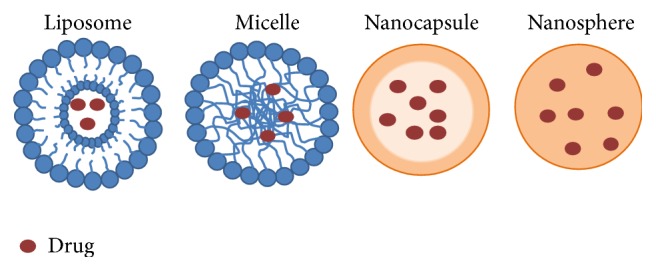
Schematic structure of liposomes, micelles, nanocapsules, and nanospheres. Liposomes are composed of one or more lipid bilayer structures surrounding an aqueous core where the drug is encapsulated. Micelles contain a hydrophilic shell and a hydrophobic core for carrying lipophilic drugs. Nanocapsules encapsulate a drug in an inner space surrounded by a polymer membrane while nanospheres are solid polymers that incorporate a drug in their matrix through uniform dispersion. Modified from [131].

Versatile novel drug delivery systems (DDS) are actively being developed to improve Vitamin E biodistribution, pharmacokinetics, and stability and subsequently raise their levels at the target site and diminish negative side effects. These systems are based mainly on three distinctive but related approaches: liposomal formulations, nanoparticles, and micellar formulations (Figure 1). Liposomes are artificially prepared self-assembled spherical vesicles composed of one or several amphiphilic phospholipid bilayers that contain an aqueous core domain. Their size ranges from 50 nm to several micrometers and can entrap both hydrophilic and hydrophobic drugs isolating them from the surrounding environment. Nanoparticles (1–100 nM) either incorporate a drug in their matrix through uniform dispersion (nanospheres) or encapsulate a drug in a cavity surrounded by a polymer membrane (nanocapsules). Polymeric micelles are comprised of a hydrophobic core, where poorly water-soluble drugs can be solubilized, and a hydrophilic shell. Their size ranges from 20 to 100 nm. Furthermore, solubilizers, such as polyethylene glycols (PEG), are also being tested as suitable formulations for human application. Besides increasing drug bioavailability, novel drug delivery systems aim to provide a synergistic effect by formulating natural Vitamin E isoforms or synthetic derivatives with other chemotherapeutic agents. This review compares and contrasts the properties and anticancer effects of the most potent Vitamin E isoforms and derivatives with emphasis on the most promising delivery approaches being developed to improve their efficacy.

## 2. Vitamin E

### 2.1. Structure and Metabolism

Vitamin E exists in nature as a group of 8 isoforms: *α*-, *β*-, *γ*-, and *δ*-tocopherols (*α*-TOC, *β*-TOC, *γ*-TOC, and *δ*-TOC, resp.) and *α*-, *β*-, *γ*-, and *δ*-tocotrienols (*α*-TT, *β*-TT, *γ*-TT, and *δ*-TT, resp.) (reviewed by [4]). Natural primary sources of tocopherols include nuts and vegetable oils, whereas tocotrienols are commonly found in palm oil, rye, oat, barley, wheat germ, and rice bran [9]. *α*-TOC is the most abundant form of Vitamin E in nature and in vitamin supplements.

**Figure 2:**
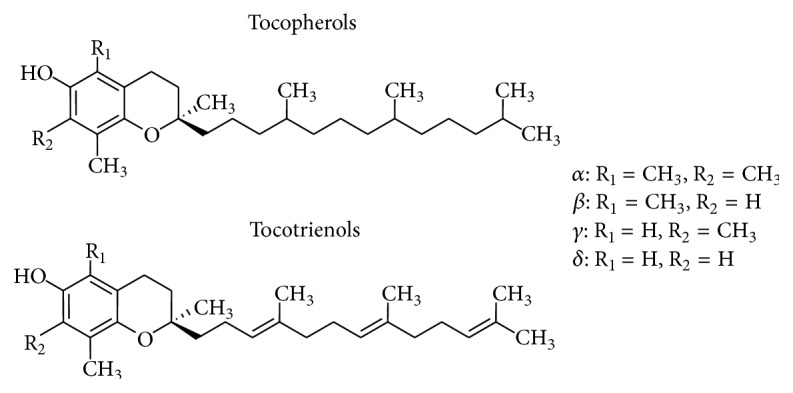
Structure of the natural isoforms of Vitamin E.

Vitamin E natural isoforms are comprised of a chroman head containing 1 phenolic acid and 1 heterocyclic ring and a phytyl tail (Figure 2). Tocopherols and tocotrienols differ in their structure: the phytyl tail of tocopherols is saturated whereas in tocotrienols it is unsaturated; the two groups also have a different number of methyl groups on the chroman head [9]. Vitamin E natural isoforms have three main moieties with distinct biological functions. (1) The first is the functional domain, composed of the redox-active hydroxyl group present in all tocopherols and tocotrienols; this is responsible for their antioxidant activity and can be modified to produce tocopheryl or tocotrienol derivatives. For example, *α*-tocopherol is esterified with a succinyl moiety to produce a-tocopheryl succinate (*α*-TOS) [10]. The functional domain is also thought to be responsible for the apoptotic properties of Vitamin E derivatives. (2) The second is the signaling domain, comprised of the aromatic rings, which regulates signaling pathways, including the protein phosphate 2/protein kinase C pathway, and (3) the hydrophobic domain, which is responsible for the binding of Vitamin E isoforms in circulating lipoproteins and biological membranes. Furthermore, the structure of the aliphatic chain may play a role in the apoptotic properties of Vitamin E isoforms, modifying membrane docking and lipid solubility [11].

Consumption of tocopherols leads to their uptake by the intestine and secretion in the circulation in chylomicron particles together with triacylglycerol and cholesterol. Chylomicron-bound Vitamin E is catabolized by lipoprotein lipase and can then be transported to peripheral tissues such as muscle, adipose, and brain [12]. The resulting chylomicron remnants are then transferred to the liver, where *α*-tocopherol transfer protein (*α*-TTP) reincorporates *α*-tocopherol into nascent very low-density lipoproteins (VLDLs). This enables further distribution of *α*-tocopherol throughout the body [13]. When the daily intake of *α*-tocopherol is excessive (over 150 mg), it is degraded to the hydrophilic *α*-CEHC (carboxyethyl-hydroxychromans, CEHC) form by a process that involves cytochrome P450 and is then primarily excreted into urine [14].

Administration of *α*-TOC and tocotrienols to healthy individuals followed by detection of their levels in plasma up to 24 h revealed that tocotrienols are bound to the triacylglycerol particles (TRPs), low-density lipoproteins (LDLs), and high-density lipoproteins (HDLs) [15]. However, the concentration of tocotrienols in the plasma was significantly lower than that of *α*-TOC. *α*-TOC was also found to be bound to HDLs and LDLs in the plasma; animals studies also suggest that *α*-TOC is more easily absorbed compared to the other natural isoforms [6, 16, 17]. The difference in the absorption of these compounds may be attributed to the number of methyl groups present on their chroman head which affects their lipophilicity and their transport through biological membranes.

It has been reported that oral administration of *α*-TOC and *α*-ΤΤ in mice led to detectable levels of both isoforms in the skin, heart, lungs, brain, liver, bone marrow, and blood suggesting that they can be effectively transported to various organs* in vivo *and that they display nonspecific distribution to tissues [18]. The cellular uptake of Vitamin E has been extensively investigated in the liver which is the major regulator of the levels of Vitamin E in the body. Evidence suggests that tocopherol can “flip-flop” out of the membranes due to its lipophilic nature [19, 20]. However, regulated transport of Vitamin E in the cell may also be facilitated by lipid transfer proteins and scavenger receptor class B type I (SR-BI) receptors (reviewed in [21]). As previously mentioned, Vitamin E isoforms are bound in the plasma to LDLs. The LDLR pathway is known to play a role in the cellular uptake of tocopherol from LDL but is not essential for the maintenance of normal tissue Vitamin E levels [22, 23]. HDL is also a major carrier of tocopherol in the plasma and contributes to its regulated cellular uptake possibly via the cubilin/megalin receptor system [24]. It has also been reported that SR-BI-mediated selective lipid uptake plays a pivotal role in the delivery of HDL-tocopherol to the central nervous system and in type II pneumocytes [25, 26]. The exact role of SR-BI in tocopherol transport in humans is still under investigation; however, SR-BI knock-out in mice hinders the transport of Vitamin E in the ovaries, testes, lung, and brain tissues [27]; interestingly, the expression of SR-BI is affected by Vitamin E status in rat liver and HepG2 cells [28].

The intracellular transport of Vitamin E has also been investigated in the liver. Following internalization in hepatocytes, Vitamin E reaches the lysosomal vesicles through the endocytic pathway. TTP facilitates intracellular transport of Vitamin E between membrane vesicles* in vitro* [29]. It has been suggested that TPP, which contains a putative lysosomal targeting motif, transiently associates with the membrane vesicle by utilizing accessory proteins to anchor to the membrane and obtains Vitamin E from the endocytic vesicles [30–33]. Functional lysosomal proteins NPC1 and NPC2 are also required for the release of tocopherol from the endosomes/lysosomes [34, 35]. At the subcellular level, Vitamin E localizes at the golgi apparatus, endoplasmic reticulum, mitochondria, and lysosomes [36].

### 2.2. Biological Properties and Anticancer Activity

Vitamin E natural isoforms display great structural homology and have similar functions such as antioxidant activity. However, studies suggest that Vitamin E isoforms also have distinct biological activities that are not common between them. For example, *α*-TOC (1) can inhibit the function of PKC, 5-lipo-oxygenase, and phospholipase in a posttranslational level and can activate phosphatase 2A and (2) can inhibit cellular proliferation, platelet aggregation, and the monocyte attachment [37]. The above functions of Vitamin E suggest that, in addition to their antioxidant properties, these isoforms can interact with enzymes, structural proteins, lipids, and transcription factors. *γ*-ΤOC has also been found to possess anti-inflammatory and antineoplastic activities [38].

Vitamin E natural isoforms and synthetic derivatives (VitE-ISDs) have been intensively investigated for their anticancer properties against several types of cancer including breast, prostate, lung, colon, gastric, and ovarian cancers and have been found to affect survival and proliferation pathways both* in vitro* and* in vivo* (reviewed by [4]). The initial evidence regarding the anticancer potential of Vitamin E was derived from studies showing that people with a diet rich in Vitamin E natural isoforms have a lower risk of colon cancer [39, 40]. Further support for the potential use of Vitamin E in cancer chemoprevention or therapy came from studies showing that dietary treatment with Vitamin E led to a reduction in the incidence of prostate cancer or a delay in its progression [41–43]. Based on the literature, *γ*-ΤOC and *δ*-ΤOC are more potent inducers of apoptosis compared to *β*- and *α*-tocopherols. Even though *α*-TOC is a potent antioxidant, accumulating evidence in the literature suggests that *α*-TOC cannot induce apoptosis [44]. Furthermore, tocotrienols are more effective proapoptotic agents than tocopherols; *γ*-ΤΤ and *δ*-ΤΤ are more potent than *α*- and *β*-forms.

The major apoptotic pathways affected by the most potent natural and synthetic Vitamin E isoforms have been extensively described in other reviews [2, 4, 45]. *γ*-ΤΤ is a well-studied natural Vitamin E isoform known to induce apoptosis via the extrinsic and intrinsic pathway, by inhibiting the NF-*κ*B transcription factor and the AKT pathway thereby lowering the levels of antiapoptotic proteins such as c-FLIP, Bcl-2, Bcl-xL, and IAPs and allowing the activation of caspases 9 and 3 [46, 47]. *γ*-ΤΤ also acts via the intrinsic pathway by inducing the translocation of Bax to the mitochondria and MOMP [48]. Vitamin E compounds have been found to induce both caspase-dependent and -independent pathways of apoptosis; however, the precise mechanism of CI-PCD induced by these compounds is still under investigation.

**Figure 3:**
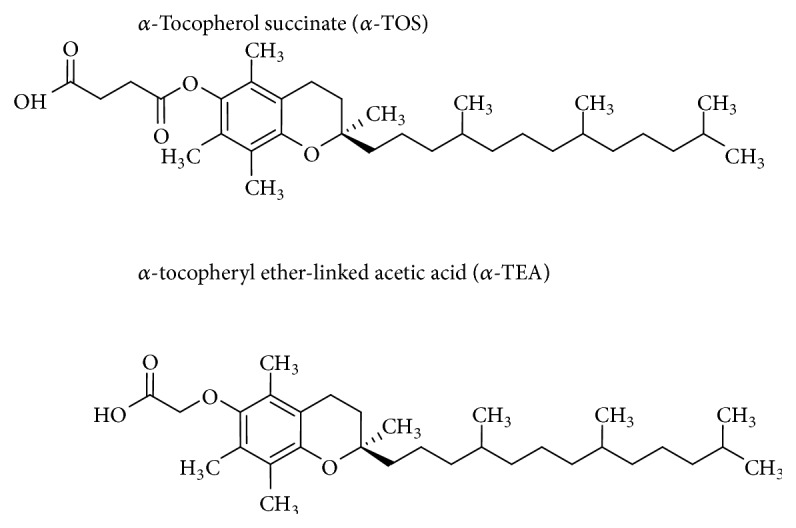
Structure of Vitamin E synthetic derivatives.

Research in the past few years has focused on structural variations within the functional moiety of natural Vitamin E isoforms with the aim of improving the proapoptotic potency of these agents and improving their bioavailability. Synthetic derivatives of *α*-TOC (Figure 3), such as *α*-tocopherol succinate (*α*-TOS) and *α*-tocopheryl ether-linked acetic acid (*α*-TEA), have shown enhanced proapoptotic potency and anticancer action in tumorigenic cell lines and animal models [49–53]. Derivative *α*-TOS is a potent apoptotic compound that acts as a mitocan; it exerts anticancer activity and selectively induces apoptosis in tumor cells mainly by targeting and destabilizing mitochondria (reviewed in [54]). *α*-TOS generates ROS, which is known to trigger the mitochondrial pathway of apoptosis, and also inactivates Bcl-2 and Bax and induces the translocation of Bax to the mitochondria [55]. *α*-TEA has been found to cooperate with chemotherapeutic agents to induce apoptosis of P53 mutant, triple negative human breast cancer cells via activating P73 suggesting that this synthetic derivative may be useful in the treatment of multidrug resistant cancers [50]. Furthermore, *α*-TEA suppressed constitutively active basal levels of p-AKT, p-ERK, p-mTOR, and their downstream targets as well as induced apoptosis in breast cancer cells [51]. *α*-TEA was also found to stimulate tumor autophagy and enhance antigen cross-presentation in murine mammary and lung cancer cells [49] and induce apoptosis via ER stress by enhancing DR5/caspase-8 proapoptotic signaling in breast cancer cells [56]. Therefore, there is great interest for further evaluation of different alpha-tocopherol synthetic derivatives in* in vitro* and* in vivo *systems either as single agents or in combination with other drugs.

## 3. Improving the Efficacy of Vitamin E Compounds with Novel Delivery Systems

The potential of Vitamin E isoforms in cancer prevention and therapy and the problems regarding their solubility and absorption have prompted the development of novel delivery systems to intensify their effects. In addition to enhancing their own anticancer effects, several drug delivery systems that incorporate Vitamin E isoforms are being developed in order to improve the efficacy of other agents, either by increasing their bioavailability or by acting in a synergistic manner. We will review below these systems that include novel formulations for both tocopherols and tocotrienols. We will present the most important preclinical studies and their applications in cancer therapeutics.

### 3.1. Tocopherol Delivery Systems

As previously described, *α*-tocopherol is esterified with a succinyl moiety to produce *α*-tocopheryl succinate or *α*-TOS. The proapoptotic properties of *α*-TOS are well known and there are numerous studies showing its enhanced anticancer potency in many cancer types including melanoma, breast, prostate, gastric, mesothelioma, and colorectal (reviewed in [4]). In addition, several formulations of *α*-TOS have been tested in order to improve its distribution and efficacy at the target site. Like other Vitamin E isoforms, *α*-TOS is subjected to intestinal hydrolysis; in order to counter this effect and to increase its uptake by rapidly divided tumor cells, high-density lipoprotein- (HDL)- associated *α*-TOS was synthesized. Tumor cells have an excessive need for cholesterol and selectively uptake HDL-associated compounds [57, 58]. *α*-TOS in association with HDL was successfully delivered to A549 lung cancer cells* in vitro *and LL2 mouse lung carcinoma cells* in vivo* via SR-BI, the prime receptor mediating selective lipid uptake from HDL, and inhibited tumor cell growth [59].

It has also been reported that analogues of Vitamin E act as mitocans; they induce apoptosis in tumor cells by affecting mitochondria stability (reviewed in [60]). To enhance this effect, a mitochondrially targeted *α*-TOS was synthesized by tagging the hydrophobic chain of the parental compound with a triphenylphosphonium group (TPP+) [61]. MitoVES was 10- to 30-fold more effective than its untagged counterpart. TPP+ has been shown to promote the selective mitochondrial uptake of antioxidant compounds driven by the mitochondrial membrane potential [62]. Conjugation of *α*-TOS with TPP+ (MitoVES) potentiated its apoptotic effect in a mitochondria-depended mechanism involving the generation of ROS and upregulation of the Noxa protein in various malignant cell lines; its effect was also potentiated in two animal cancer models (Table 1) [61]. In a different study, MitoVES induced apoptosis in mesothelioma cells more efficiently than *α*-TOS by destabilizing the mitochondrial membrane potential and generating ROS. The mitochondrially targeted *α*-TOS also suppressed mesothelioma growth in nude mice with high efficacy [63]. MitoVES selectively suppressed the proliferation of cancer cells at subapoptotic doses by affecting mitochondrial DNA (mtDNA) transcripts. Specifically, MitoVES strongly suppressed the level of the displacement loop transcript and of mtDNA genes coding for subunits of mitochondrial complexes causing disruption of mitochondrial function. In addition, MitoVES decreased the expression of mitochondrial transcription factor A (TFAM) and diminished mitochondrial biogenesis. Importantly, the inhibition of mitochondrial transcription was replicated* in vivo*; MitoVES lowered the level of mtDNA transcripts in HER2 overexpressing breast cancer cells but not in normal tissue [64].

Due to its amphipathic structure, *α*-TOS forms vesicles in aqueous solutions (TS-NVs) and may be used as a prospective tool for the encapsulation and delivery of other drugs. However, *α*-TOS vesicles are unstable in the presence of divalent cations, acidic pH, and serum. In order to improve their stability, a novel nanovesicle consisting of TS and egg phosphatidylcholine (TS-EPC-NVs) was synthesized. TS-EPC-NVs, at a concentration ranging from 12.5 to 50 *μ*M, displayed greater stability and more potent* in vivo* and* in vitro *anticancer effects (the* in vitro* anticancer efficiency increased sevenfold) than that of TS-NVs that were attributed to a more effective intratumoral distribution, homogenous cellular uptake, and enhanced cytosolic delivery [65]. The TS-EPC-NVs showed enhanced anticancer effects without the encapsulation of a drug, highlighting the potential usefulness of Vitamin E in cancer.

Vitamin E analogues can be easily incorporated into lipid bilayers to produce liposomes of various surface modifications and size distributions. Liposomes can encapsulate a single drug for monotherapy or several drugs for multitargeted effects. The drugs incorporated into liposomes are passively delivered to tumor tissue by enhanced permeation and retention (EPR) effect. The EPR effect is the property by which certain molecules accumulate in tumors rather than in normal tissues, due to the presence of more blood vessels in tumor tissue [66, 67]. In order for tumor cells to grow quickly, they stimulate the production of blood vessels for their nutritional and oxygen supply. These newly formed tumor vessels are usually abnormal in form and architecture which leads to abnormal molecular and fluid transport dynamics, especially for macromolecular drugs. Thus, the EPR effect helps to carry micelles, nanoparticles, and liposomes and spread them inside the cancer tissue.

A derivative of *α*-TOC, a-tocopheryl maleamide (*α*-TAM), contains a noncleavable amide bond and has exhibited enhanced apoptotic properties* in vitro* [68]. In a liposomal formulation, *α*-TAM induced apoptosis in human and murine cancer cells* in vitro* and suppressed tumors in mouse models. Importantly, *α*-TAM's nonspecific toxicity and immunotoxicity were diminished when incorporated into liposomes [69]. Another derivative of Vitamin E with potent anticancer activity, *α*-TEA, in combination with 9-nitrocamptothecin (9-NC), a derivative of camptothecin, was formulated into liposomes using dilauroylphosphatidylcholine and administered by aerosol in tumor-bearing mice. Liposome-formulated *α*-TEA and 9-NC significantly reduced the growth, induced apoptosis, and inhibited metastasis of mouse mammary gland cell line (66 cl-4-GFP). Treatment of 66 cl-4-GFP cells in culture for 3 days with a combination of *α*-TEA (10 mg/mL, singly produces 38% apoptosis) and 9-NC (15.6, 31.3, 62.5, or 125 ng/mL; singly produces 2–7% apoptosis) produced 47%, 58%, 64%, and 69% apoptosis. In control animals, the incidence of macroscopic lung metastasis was 83% compared to 8% in *α*-TEA-, 9-NC-, or combination-treated mice [70]. It was calculated that approximately 36 g *α*-TEA and 0.4 g 9-NC per mouse per day were deposited in the lungs, respectively.

Micelles are commonly used as a platform for the delivery of hydrophobic drugs. They display good solubilization efficiency and high stability upon dilution and their nanoscale dimensions permit the efficient accumulation in tumor tissues via the EPR effect [71–73]. Chitosan is widely used in micellar drug delivery applications due to its excellent biocompatibility, nontoxicity, biodegradability, and low immunogenicity. *α*-TOS has been synthesized in combination with paclitaxel (PTX) in chitosan derivative polymeric micelles for cancer cell delivery. This conjugate self-assembled in aqueous medium to form micelles and PTX was incorporated into the micellar core for intravenous delivery. Commonly, PTX is diluted in a 50 : 50 mixture with Cremophor EL which has severe toxic side effects. A polymeric micelle system of *α*-tocopherol succinate-amphiphilic chitosan (CS-TOS) loaded with PTX displayed comparable cytotoxicity to PTX-Cremophor EL and free PTX against MCF-7 cells* in vitro* and significantly inhibited the growth of U14 tumors* in vivo* at doses of 10 and 20 mg/kg with no toxic side effects [74]. This further highlights the usefulness of *α*-tocopherol succinate in replacing harmful lipophilic solvents. In an aim of improving the therapeutic efficiency and reducing side effects of PTX, *α*-TOS-grafted chitosan oligosaccharide was synthesized and physically loaded by PTX and *α*-TOS. The incorporation of *α*-TS into the micelles led to an increase of the hydrophobic interaction between PTX and the micelles core, thereby improving micelle stability, reducing micelle size, and facilitating PTX release from the micelles. In addition, *α*-TS/PTX-loaded micelles displayed higher cytotoxicity against human ovarian cancer cells* in vitro* than that of PTX-loaded micelles and PTX alone [75].

*α*-TOS has also been used as a hydrophobic scaffold for a novel molecular platform, the SS-cleavable Proton-Activated Lipid-like Material (ssPalm). This pDNA-encapsulating nanoparticle was designed to degrade in the cytosolic environment. The lipid envelope coating the condensed DNA/polycation complex was a stable bilayer composed of the ssPalm molecule which mounts dual sensing motifs that respond to various intracellular environments and allow for the release of the encapsulated molecule in the cytoplasm [76]. ssPalm contains a proton-sponge unit (tertiary amines) that functions in response to an acidic environment, such as in an endosome or lysosome, and disulfide bonding that can be cleaved in a reducing environment such as the cytosol. ssPalmE, where Vitamin E (*α*-tocopherol succinate) was used as a hydrophobic scaffold, successfully delivered a solute form of VEGFR* in vivo* and showed enhanced antitumor action in mice bearing tumors established from a renal cell carcinoma (OS-RC-2). The ssPalmE formulation was significantly more effective than ssPalmM, where Myristic acid was the hydrophobic scaffold, suggesting that *α*-TOS worked synergistically with the VEGFR agonist against renal cell carcinoma* in vivo *[77]. LNPssPalmM and LNPssPalmE were administered intravenously at a dose of 37.5 *μ*g of pDNA/mouse 3 times at every 3 days.

### 3.2. Tocotrienol Delivery Systems

Novel DDS aim to target compounds to tumor sides thereby diminishing side effects on healthy tissues. To achieve specific distribution to tumors, tocotrienol extract from palm oil or tocotrienol-rich fraction (TRF) was encapsulated within vesicles bearing transferrin (Table 2). The transferrin receptor is a cell membrane-associated glycoprotein involved in iron homeostasis and the regulation of cell growth; since transferrin receptors are overexpressed up to 100-fold in several types of cancer cells compared to normal cells, these vesicles could selectively localize to tumors. This novel formulation of TRF led to a 3-fold higher uptake and improved its toxicity more than 100-fold in A431 (epidermoid carcinoma), T98G (glioblastoma), and A2780 (ovarian carcinoma) cell lines compared to TRF solution. The vesicle formulations of TRF were all significantly more effective than the free drug by at least 80 times, with IC_50_ ranging from 0.05 ± 0.02 to 1.42 ± 0.30 *μ*g/mL depending on the cell line. Intravenous administration of transferrin-bearing vesicles loaded with TRF induced tumor regression and improvement of animal survival in a murine xenograft model and was well tolerated by animals [78]. However, tumor regression lasted only for the duration of the treatment. To improve the therapeutic efficacy, TRF was encapsulated in multilamellar rather than unilamellar transferrin-bearing vesicles. Multilamellar vesicles contain more than one phospholipid bilayers compared to unilamellar; this may improve tocotrienol loading within the lipidic membranes. This novel formulation not only significantly improved tocotrienol uptake by transferrin-expressing tumor cells but also improved the* in vitro* therapeutic efficacy of free tocotrienol from 17- to 72-fold depending on the cell line. Importantly, this novel TRF formulation led to complete tumor suppression for 40% of B16-F10 murine melanoma tumors and 20% of A431 human epidermoid carcinoma tumors, with long-term survival of the animals [79].

In addition to their inherent anticancer properties, tocotrienols can also potentiate the anticancer activity of other drugs thereby lowering their effective concentration and limiting toxicity. Tocotrienols can enhance the effect of several compounds including cox-2 inhibitor celecoxib, tyrosine kinase inhibitor gefitinib, and statins such as Simvastatin [80–82]. Simvastatin is a potent inhibitor of 3-hydroxy-3-methylglutaryl-coenzyme A (HMGCoA) reductase that displays anticancer activity but its clinical use is limited by high-dose toxicity. In an effort to produce bioactive, injectable nanoparticles that carry both TRF and Simvastatin, parenteral lipid nanoemulsions loaded with both compounds were synthesized; nanoemulsions are comprised of nanoscale droplets in the range of 1–100 nm and have many applications in pharmaceutics (reviewed in [83]). Lipid nanoemulsions containing Simvastatin at subtherapeutic doses decreased the IC_50_ of TRF in MCF-7 (from 14 to 10 *μ*M) and MDA-MB-231 (from 7 to 4.8 *μ*M) breast cancer cells [84]. Different nanoemulsions for optimized incorporation of tocotrienol- (T3-) rich palm oil or Tocomin obtained with different homogenization strategies have also been tested for their anticancer activities. Adopted hybrid nanoemulsification of Tocomin (Tocomin-NE) displayed 2,2-diphenyl-1-picrylhydrazyl- (DPPH-) radical scavenging capacity which effectively permeated cell membranes by diffusion and demonstrated significantly stronger cytotoxic profiles (at least 5-fold lower IC_50_ values, compared to those estimated for the Tocomin-control). This hybrid nanoemulsified formulation of T3-rich palm oil may be used in topical delivery against skin carcinomas [85].

Tocotrienols have also been formulated with Epirubicin (EPI) to reduce its toxicity. EPI is an anthracycline derivative used commonly in the treatment of Hepatocellular carcinoma (HCC) but displays serious side effects including cardiomyopathy and congestive heart failure. To specifically target hepatocytes, EPI was loaded in chitosan-PLGA nanoparticles linked with asialofetuin (EPI-NPs); furthermore, to reduce cardiotoxicity, targeted EPI-NPs were coadministered with tocotrienols. Combined therapy of tocotrienols with EPI-NPs enhanced apoptosis, reduced VEGF level in a dose dependent manner, and provided protection against oxidative stress and inflammation induced by EPI in the heart [86].

## 4. D-Alpha-Tocopheryl Polyethylene Glycol Succinate (TPGS)

### 4.1. Physicochemical Properties

D-alpha-tocopheryl polyethylene glycol succinate (Vitamin E TPGS or TPGS) is a synthetic derivative of natural alpha-tocopherol which is gaining increasing interest in the development of drug delivery systems (reviewed by [87]). TPGS, prepared from the esterification of *α*-TOS and polyethylene glycol (PEG) 1000, possesses the advantages of PEG and Vitamin E in drug delivery applications, including the ability to extend the half-life of the drug in the plasma.

**Figure 4:**
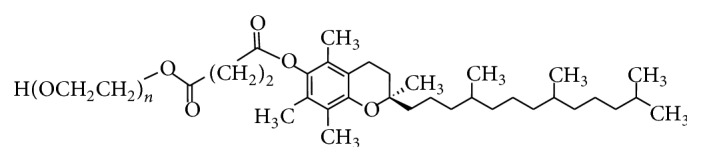
D-alpha-tocopheryl polyethylene glycol succinate structure. TPGS is synthesized from the esterification of *α*-TOS and PEG 1000. Modified from [104].

The PEG 1000 has an average molecular weight of about 1000 Dalton and is synthesized from fossil fuel while alpha-tocopherol succinate is produced by the alpha-tocopherol which is esterified to succinic acid. TPGS has an average molecular weight of 1513 and an amphiphilic structure of lipophilic alkyl tail and hydrophilic polar head; the PEG 1000 portion of the molecule is water soluble, while the alpha-tocopherol portion is fat soluble [87]. Structurally, TPGS is very similar to the tocopherols; it has a phytyl tail and a chroman ring but the hydroxyl group on the chroman head is esterified to the polyethylene 1000 succinate moiety (Figure 4). The molecular formula is C33 O5 H54 (CH_2_ CH_2_ O)*n*, where “*n*” represents the number of polyethylene oxide moieties attached to the acid group of alpha tocopheryl succinate.

TPGS is a waxy solid that completely dissolves in water and forms its own micelles; the hydrophilic regions of the molecule are in contact with the surrounding solvent, sequestering the hydrophobic tail regions in the micelle centre. It is also miscible with oils, other surfactants, and cosolvents. TPGS has a melting point around 37–41°C and is heat stable at temperatures up to 199°C [88]. This is very important for applications in the pharmaceutical industry because it can be processed at thermally stable temperatures without degradation. TPGS is stable at pH 4.5–7.5; however, it degrades in alkaline or highly acidic environments where the ester linkages are hydrolyzed [88].

TPGS was developed in the 1950s by Eastman Chemical Company (Kingsport, TN) as a water-soluble form of Vitamin E in order to be administered to individuals who cannot absorb naturally occurring lipophilic alpha-tocopherol. These include patients with cystic fibrosis, Crohn's disease, short bowel disease, pancreatic enzyme deficiency, or cholestatic liver disease [89–91]. Importantly, the United States' Food and Drug Administration (FDA) as well as the European Food Safety Authority (EFSA) has already approved TPGS as a safe pharmaceutical adjuvant used in drug formulation and has estimated the safety limits for TPGS use for research purposes [92, 93]. It has been reported that the acute oral median lethal dose (LD_50_), which is defined as the quantity of an agent that will kill 50 percent of the test subjects within a designated period, is >7 g/kg for young adult rats of both sexes [94]. Following studies both in humans and in animals to address the bioavailability and safety of TPGS, the EFSA concluded that the overall no-observed-adverse-effect level (NOAEL) of TPGS is 1000 mg/kg bw/day. In addition, TPGS was found to be nongenotoxic [95].

The mechanism of TPGS to enter the enterocyte and become absorbed has also been investigated: TPGS may be hydrolyzed to free alpha-tocopherol in the stomach by nonenzymatic hydrolysis, in the proximity of the brush border epithelium by esterase hydrolysis, and on the surface of the enterocytes via a lipase or entire TPGS micelles can pass through cell membranes thereby enabling the absorption of the intact TPGS molecule [96].

In recent years, TPGS has been intensively applied in developing various drug delivery systems as an absorption enhancer, emulsifier, solubilizer, additive, permeation enhancer, and stabilizer. The coadministration of TPGS has been shown to enhance drug solubility, to inhibit P-glycoprotein (P-gp) mediated multidrug resistance (MDR), and to increase the oral bioavailability of anticancer drugs [97–100]. As an effective emulsifier, TPGS has been shown to greatly enhance the performance of nanoparticles, resulting in much higher cellular uptake of the drug as well as more desirable* in vivo* pharmacokinetics [101–103]. Importantly, emerging evidence in the literature suggests that TPGS not only may be useful in cancer chemotherapy as a carrier drug but also may act synergistically or enhance the effect of anticancer drugs.

### 4.2. Anticancer Activity as a Single Agent

As a single agent, TPGS has been found to inhibit the growth of human prostate and lung carcinoma cells both* in vitro* and* in vivo *[105, 106]. TPGS inhibited the growth of A549 human lung carcinoma cells* in vivo* and, in an* in vitro* cell culture, more potently than *α*-TOS. Compared to *α*-TOS, TPGS was more effective at inducing apoptosis in A549 cells as measured by DNA fragmentation; furthermore, at 40 *μ*M concentration, TPGS induced the production of ROS [105]. Since PEG conjugation may affect the rate of cell uptake of drugs, the time-dependent accumulation of *α*-TOS and TPGS in H460 cells was measured. However, the time-dependent uptake of TPGS into cells did not differ from that of *α*-TOS. Furthermore, since TPGS may be hydrolyzed after entering the cells, the intracellular concentration of TPGS was measured following treatment for up to 8 h and no detectable levels of *α*-TOS were found [105]. This indicates that TPGS is not rapidly hydrolyzed to *α*-TOS and that the enhanced antitumor efficacy of TPGS was not due to its increased uptake into cells.

The ability of TPGS to induce apoptosis was also investigated in androgen receptor negative (AR−) DU145, PC3, and androgen receptor positive (AR+) LNCaP prostate cancer cells [106]. TPGS induced caspase-3 activity solely in the AR+ LNCaP cells; however, TPGS induced dominant caspase-independent programmed cell death (CI-PCD) in all cell lines examined, suggesting that the molecular pathways being induced by the compound may depend on the cellular microenvironment. In a recent study conducted by our group, TPGS at 20 *μ*M induced G1/S cell cycle arrest and apoptosis in breast cancer cell lines MCF-7 and MDA-MB-231. An investigation of the molecular mechanism of action of TPGS revealed that induction of G1/S phase cell cycle arrest is associated with upregulation of P21 and P27Kip1 proteins. Induction of apoptosis by TPGS involved the inhibition of phospho-AKT and the downregulation of the antiapoptotic proteins Survivin and Bcl-2 [107]. Importantly, TPGS did not reduce the viability or proliferation of “normal” (nontumorigenic) immortalized cells (MCF-10A and MCF-12F) suggesting that its effects are cancer cell-specific.

### 4.3. Applications in Drug Delivery Systems

TPGS has been used in various drug delivery systems as a solubilizer/absorption enhancer, as a permeation enhancer, and as a potent P-gp inhibitor. Solubilizers/absorption enhancers are functional agents included in formulations to increase the solubility or improve the absorption of a substance. TPGS can increase the solubility of drugs such as cyclosporines, taxanes, steroids, and antibiotics by incorporation into TPGS micelles [108]. TPGS has been used as a solubilizer for PTX, celecoxib, corticosteroids, capuramycin analog SQ641, and Propofol (reviewed by [88]). Furthermore, TPGS has been shown to increase the absorption flux of HIV protease inhibitor Amprenavir and to enhance the bioavailability of cyclosporine in humans and of Colchicine in rats [109, 110]. TPGS has also been used as a permeation enhancer; it is incorporated into formulations to promote their permeation through the skin or intestinal walls. TPGS was found to enhance the permeability of Amprenavir and Colchicine and has potential as an enhancer of drug permeability in colonic tissue [88, 111].

TPGS has also been used in combination with other compounds due to its ability to inhibit P-gp, an ATP-dependent drug efflux pump, also known as multidrug resistance protein 1 (MDR1) or ATP-binding cassette subfamily B member 1 (ABCB1). P-gp is responsible for the transport of a wide variety of substrates across extracellular and intracellular membranes. It is widely expressed in hepatocytes, the renal proximal tubular cells, intestinal epithelium, adrenal gland, and capillary endothelial cells comprising the blood-brain and blood-testis barrier. P-gp is often overexpressed in cancer cells and confers MDR by decreasing drug accumulation of chemotherapeutic drugs such as PTX, Etoposide, Doxorubicin (DOX), and Vinblastine. TPGS can inhibit the P-gp ATPase (P-gp energy source of active transport) by binding to the nontransport active binding site, resulting in inhibition of P-gp mediated drug transport and multidrug resistance [99, 112]. Specifically, combination of TPGS with DOX, Vinblastine, PTX, or Colchicine was found to enhance their cytotoxicity in NIH 3T3 cells transfected with the human MDR1 cDNA [99]. In a different study, TPGS and high concentrations of polysorbate 80 inhibited* in vitro* both efflux transporters, ABCB1 (P-gp) and ABCC2 (MRP2), that play an essential role in the limitation of oral bioavailability of drugs [113]. So far, TPGS synthesized with PEG-1000 is the most potent efflux pump inhibitor [114]. Furthermore, TPGS can inhibit cytochrome P450 3A (CYP3A) that has been associated with deactivation of several anticancer drugs [115].

Recent studies suggest that the coadministration of TPGS with chemotherapeutic agents may overcome drug resistance by increasing their cellular entry and retention and by acting in a synergistic manner (Table 3). The addition of TPGS (0.03% (w/w)) in a nanocarrier loaded with DOX decreased P-gp activity in MCF-7/ADR cells, increased the nuclear accumulation of the drug, and increased the therapeutic efficacy against the resistant cell line [116]. Cells were treated with free Dox (from 0.13 *μ*M to 10 *μ*M) and TPGS-coated porphyrin-polylactide nanoparticles (PPLA-NPs) or TPGS-coated Dox-PPLA-NPs (from 0.07 *μ*M to 4.3 *μ*M). When the PPLA-NPs were coated with TPGS, cell viability was significantly reduced even at the lowest concentrations [116]. In an alternative approach to overcome resistance in MCF-7/ADR cells, DOX was loaded in mitochondria-targeted pH-responsive (PDPA) micelles containing TPGS. In the acidic pH of early endosomes, the DOX payload was released while the TPGS component synergistically improved the cytotoxicity of the agent by reducing the mitochondrial transmembrane potential. PDPA/TPGS micelles reduced the IC_50_ of DOX in MCF-7/ADR cells by a sixfold magnitude [117]. A TPGS-mixed micelle system loaded with Resveratrol has also been formulated. Resveratrol has shown efficacy in overcoming chemoresistance in breast cancer but its effects are hindered by poor absorption and rapid metabolism. Resveratrol-loaded mixed micelles composed of methoxy poly (ethylene glycol)-b-polycaprolactone (mPEG-PCL) and TPGS increased Resveratrol uptake by MCF-7/ADR cells and induced higher rates of apoptosis. Blank mixed micelles did not affect the proliferation of MCF-7/ADR cells (<10% inhibition), whereas Resveratrol alone (0–100 *μ*M) inhibited the proliferation of MCF-7/ADR cells in a dose dependent manner. Resveratrol-loaded mixed micelles enhanced the cytotoxicity of Resveratrol against MCF-7/ADR cells at all of the tested concentrations. Furthermore, Resveratrol-loaded mixed micelles inhibited the activity of P-gp and significantly enhanced DOX cellular accumulation and cytotoxicity in MCF-7/ADR cells [118].

TPGS has also been used in several formulations in order to achieve targeted delivery of chemotherapeutic drugs and reduce negative side effects. Recently, TPGS-based copolymers were synthesized, conjugated with Herceptin for targeted delivery of anticancer drugs such as Docetaxel (DTX) to cancer cells overexpressing the HER-2 receptor. Docetaxel was used at concentrations ranging from 0.025 to 2.5 *μ*g/mL. Among the nanoparticles synthesized, those with chain length PEG 1000 resulted in the best therapeutic effects. Compared to the formulations with chain lengths 2000, 3350, and 5000, the IC_50_ values of the 1000-chain polymer were 68.1%, 90%, and 92.6% lower, respectively [119]. TPGS-coated liposomes enhanced cellular uptake and cytotoxicity of DTX in brain cancer cells [120], while targeted delivery of DTX by transferrin-conjugated TPGS micelles led to significantly higher cellular uptake and higher cytotoxicity against MDA-MB-231 cells* in vitro* and reduced tumor size in xenograft SCID mice [121]. Based on the IC_50_ values, the nontargeted and targeted micelles could be 15.31- and 71.73-fold more effective than DTX after 24 h treatment. Furthermore, a TPGS-Cisplatin micellar formulation not only showed significant enhancement of Cisplatin cytotoxicity in HepG2 hepatocarcinoma cells but also showed important neuroprotective effects [122]. SH-SY5Y neuroblast-like cells, a common model for neurotoxicity test, were not greatly affected by treatment with the TPGS-Cisplatin micelle in contrast to Cisplatin alone. Therefore, it was suggested that TPGS supplementation should be adopted in patients receiving Cisplatin-based chemotherapy to reduce the neurotoxicity. In an effort to reduce the negative side effects of PTX, TPGS was used as an emulsifier in Cremophor EL-free PTX-loaded poly(lactic-co-glycolic acid)- (PLGA-) mPEG nanoparticles (NP-PTX). This novel NP-PTX formulation consisting of 160.1 mg PLGA-mPEG, 40.2 mg PTX, and 0.035% TPGS displayed similar potency as free PTX in ovarian cancer both* in vivo *and* in vitro *and 10-fold less toxicity with no organ damage (NP-PTX LD_50_ 246.85 mg/kg, free PTX LD_50_ 23.58 mg/kg) [123]. The efficacy of PTX encapsulated in TPGS-emulsified polymeric nanoparticles (TENPs) was also examined in A549 lung cancer cells at a concentration ranging from 0.01 to 50 mM. TPGS was used as an emulsifier to facilitate nanoparticle formation in an ethanol-water system at a concentration of 0.6%. The PTX-TENPs preferentially accumulated in tumors of A549 lung cancer xenograft models and significantly inhibited tumor growth following intravenous injection [124].

As previously mentioned, due to its physicochemical properties, TPGS has emerged as an ideal platform for the enhanced delivery of poorly soluble drugs. PTX and DTX are commonly used in several types of cancer and display high efficacy but they are highly lipophilic. To enhance their solubility, PTX and DTX were encapsulated in a mixed dendrimer-TPGS formulation. Dendrimers are nanomaterials that carry the guest molecule either by attachment to its surface or by encapsulation in the interior void spaces. Cells were incubated with the different concentrations (2.5–500 nM) of dendrimer-drug formulations along with free PTX and DTX for 48 h. The solubility and anticancer activities of both compounds were greatly enhanced after encapsulation in micelles against MCF-7 and A549 cancer cells and caused very low toxicity to CHO normal ovarian cells [125]. TPGS-poly(lactic acid) (TPGS-PLA) micelles have been used for the simultaneous delivery of a novel triple drug combination, antitumor drugs, Crizotinib and Palbociclib, combined with Sildenafil, an agent that increases drug accumulation in the intracellular compartment. TPGS-PLA copolymers encapsulated the three drugs with high loading efficiency and exhibited an improved cytotoxic effect in comparison with single (Crizotinib) or dual (Crizotinib-Palbociclib) formulations in A549 lung cancer cells [126].

A TPGS derivative with a PEG 2000 chain (TPGS-2k) was found to act synergistically with DTX to reduce the viability of MCF-7 cells [127]. TPGS-2k has also been used for the stabilization of a Doxorubicin-*α*-TOS conjugate. *α*-TOS was connected to Doxorubicin through an amide bond to form N-Doxorubicin-*α*-D-tocopherol succinate (N-DOX-TOS). In aqueous solutions in the presence of TPGS-2k, this formulation self-assembled into 250 nm nanostructures and localized to the core of the nanoparticle. The N-DOX-TOS nanoparticles significantly reduced the growth of MCF-7 cells and induced a higher delay in tumor growth in CT26-tumor-bearing mice compared to the control group [128]. In support of the specific anticancer properties of TPGS, a recent study revealed that blank TPGS nanoparticles showed toxicity against A549 lung cancer cells without harming normal cells [129]. In a nanoparticle formulation, TPGS further enhanced the toxicity of Morin Hydrate (MH), a naturally occurring bioflavonoid, against A549 cells both* in vitro* and* in vivo *[129].

## 5. Conclusions 

In conclusion, recent studies have highlighted Vitamin E isoforms and especially TPGS as ideal molecular biomaterials in developing various drug delivery systems, including micelles, nanoparticles, and liposomes. DDS serve to improve the delivery of Vitamin E isoforms so they can act as single agents against tumor cells or can be used as a platform for the combined treatment of Vitamin E with chemotherapeutic drugs. Vitamin E-combined therapy represents an intriguing therapeutic strategy for common chemotherapeutics, providing not only superior efficacy but also higher safety levels. TPGS has a multifunctional nature; it can act as an anticancer agent specifically against malignant cells but may also be used in nanomedicine formulations in order to increase the bioavailability and limit of the toxicity of other agents. Based on its mechanism of action, TPGS-based DDS can be formulated to combine the effect of compounds with different activity in order to achieve simultaneous targeting of parallel pathways or sequential steps in the same pathway in cancer cells and provide additive or synergistic effects. For example, the ability of TPGS to induce apoptosis in triple negative breast cancer cells (TNBC) via the PI3K/AKT pathway [107] may serve as the basis for the development of a TPGS-based nanoparticle loaded with pharmacological inhibitors of PI3K or AKT; studies show that PI3K inhibition impairs BRCA1/2 expression and sensitizes cells to PARP inhibition in TNBC [130]. Therefore, for a synthetic lethality effect, this nanoparticle may be loaded with a specific PARP inhibitor that has also been found to be highly effective in TNBC cells. Further characterization of Vitamin E isoforms' properties and mechanisms of action will provide a new insight into the design of novel Vitamin E-based chemotherapeutics and allow the development of a series of cancer-cell-selective therapies.

## Figures and Tables

**Table 1:** Novel formulations to improve the delivery and anticancer effect of tocopherols alone and in combination with other drugs.

Formulation	Cancer model	Effect	IC_50_	Reference
*α*-TOS-HDL	A549 lung cancer cells *in vitro*, LL2 mouse lung cancer *in vivo *	High capacity of *α*-TOS uptake via the SR-BI receptor, inhibition of growth	7 *μ*g *α*-TOS/25 *μ*g HDL-protein/mL medium	[59]

*α*-TOS-TPP+ (MitoVES)	T-cell lymphoma, mesothelioma, breast, colorectal, lung, and cervical cancer, neuroblastoma, FVB/N c-neu mice carrying the rat HER-2/neu proto-oncogene, and Balb c mice injected with colorectal HCT116 cells	Robust apoptosis due to mitochondrial targeting of *α*-TOS and production of ROS, suppression of tumors	MitoVES IC_50_ ranging from 0.48 to 21 *μ*M depending on the cell line	[61]

*α*-TOS-TPP+ (MitoVES)	Mesothelioma cells *in vitro* and *in vivo *	Mitochondrial destabilization, loss of mitochondrial membrane potential, generation of ROS, destabilization of respiratory supercomplexes, and suppression of mesothelioma growth in nude mice	MitoVES IC_50_ ranging from 0.25 to 2 *μ*M depending on the cell line	[63]

TS-EPC-NVs	B16-F1 mouse melanoma cells *in vitro* and *in vivo *	Homogenous cellular uptake, enhanced cytosolic delivery and effective intratumoral distribution, induction of apoptosis *in vitro*, and suppression of tumor growth	N/A	[65]

Liposomal formulation of *α*-TAM	MCF-7 or B16F10 cells implanted in the peritoneum of Balb/c mice, transgenic FVB/N c-neu mice bearing spontaneous breast carcinomas	Inhibition of proliferation of cancer cells *in vivo* and suppression of breast carcinomas	13.3 and 5.2 *μ*M for MCF-4 and B16F10 cells, respectively	[69]

Liposomal formulation of *α*-TEA and 9-NC	Mouse mammary gland cell line 66 cl-4-GFP in Balb/c mice	Inhibition of tumor growth and metastasis	N/A	[70]

Micelle system of *α*-TOS-CS-PTX	MCF-7 cells *in vitro*, U14 cervical cancer cells in Kunming mice	Cytotoxicity *in vitro*, inhibition of tumor growth	N/A	[74]

Micelle system of *α*-TOS-CS-PTX	Human ovarian cancer cells *in vitro *	Improved micelle stability and PTX release, increased cytotoxicity	110 and 188 ng/mL PTX-loaded micelles modified and unmodified with *α*-TOS, respectively	[75]

Nanoparticle ssPalmE loaded with VEGFR	Renal cell carcinoma (OS-RC-2-bearing mice)	Successful delivery of VEGFR and significant suppression of tumor growth	N/A	[77]

High-density lipoprotein (HDL), triphenylphosphonium group (TPP+), TS (*α*-TOS), egg phosphatidylcholine (EPC), nanovesicle (NV), chitosan (CS), paclitaxel (PTX), and SS-cleavable Proton-Activated Lipid-like Material Vitamin E (ssPalmE).

**Table 2:** Novel formulations to improve the delivery and anticancer effect of tocotrienols alone and in combination with other drugs.

Formulation	Cancer model	Effect	IC_50_	Reference
Unilamellar TRF-vesicles bearing transferrin	A431 (epidermoid carcinoma), T98G (glioblastoma), and A2780 (ovarian carcinoma) cells, A431 cells implanted in BALB/c mice	Threefold higher TRF uptake and more than 100-fold improved cytotoxicity *in vitro*, tumor regression, and improvement of animal survival	Ranging from 0.05 ± 0.02 to 1.42 ± 0.30 *µ*g/mL depending on the cell line	[78]

Multilamellar TRF-vesicles bearing transferrin	A431 human epidermoid carcinoma, T98G human glioblastoma, B16-F10 mouse melanoma cells, and A431 or B16-F10-luc-G5 tumors in BALB/c mice	Improved TRF uptake and cytotoxicity *in vitro*, slower growth of A431 and B16-F10 tumors, and long-term survival of 100% of the animals	Ranging from 0.89 ± 0.11 to 4.09 ± 0.65 *μ*g/mL depending on the cell line	[79]

Lipid nanoemulsions loaded with TRF and Simvastatin	MCF-7 and MDA-MB-231 breast cancer cells	Decrease of TRF IC_50_	Decreased from 14 to 10 *μ*M in MCF-7 and from 7 to 4.8 *μ*M in MDA-MB-231 cells when Simvastatin was added	[84]

Nanoemulsified formulation of T3-rich palm oil (Tocomin-NE)	Human cutaneous carcinoma *in vitro *	Increased cytotoxicity	Tocomin-NE 42.6 ± 3.8 mM and 47.3 ± 3.2 mM, Tocomin control 217.4 mM and 278.5 mM in A431 cells and SCC-4 cells, respectively	[85]

EPI-NPs coadministered with tocotrienols	Hep G2 (HCC) cells *in vitro*, HCC mouse model in Albino mice	Enhanced antiproliferative effect *in vitro*, enhanced apoptosis, and reduced VEGF level *in vivo *	Free EPI viability >90% EPI-NPs 0.9 *μ*g/mL, EPI-NPs tocotrienols 2 *μ*g/mL	[86]

Tocotrienol-rich fraction (TRF), Epirubicin (EPI), nanoparticles (NPs), and Hepatocellular carcinoma (HCC).

**Table 3:** TPGS-based delivery systems of anticancer drugs.

Formulation	Drug	Cancer type	Effect	IC_50_	Reference
Nanoparticle	DOX	MCF-7/ADR cells *in vitro *	Decreased P-gp activity, increased drug nuclear accumulation, and increased therapeutic efficacy	N/A	[116]

Mitochondria-targeted pH-responsive micelles	DOX	MCF-7/ADR cells *in vitro* and *in vivo *	Reduced mitochondrial transmembrane potential, synergistic cytotoxicity	DOX 73.2 *μ*g/mL, PDPA-TPGS-DOX 16.7 *μ*g/mL	[117]

TPGS-mPEG-PCL micelles	Resveratrol	MCF-7/ADR cells *in vitro *	Increased Resveratrol uptake, enhanced apoptosis, and inhibition of P-gp activity	N/A	[118]

TPGS copolymers conjugated with Herceptin loaded with DTX	DTX	SK-BR-3 cancer cells	Targeted delivery, efficient cellular uptake, and improved cytotoxicity	Nanoparticles without Herceptin 3.29 *μ*g/mL and with Herceptin 0.341 *μ*g/mL	[119]

TPGS coated liposomes of DTX	DTX	C6 glioma cells	Enhanced cellular uptake and cytotoxicity	DTX alone 37.04 ± 1.05, TPGS coated liposomes 5.93 ± 0.57 *μ*g/mL	[120]

Transferrin-conjugated TPGS micelles	DTX	MDA-MB-231 cells *in vitro* and in xenograft SCID mice	Targeted delivery, higher cellular uptake, higher cytotoxicity, and reduced tumor size	DTX alone 13.63 ± 0.12, nontargeted micelles 0.89 ± 0.10, and targeted micelles 0.19 ± 0.04 *μ*g/mL	[121]

TPGS micelle	Cisplatin	HepG2 hepatocarcinoma cells	Enhanced cytotoxicity, neuroprotective effects	Cisplatin alone 3.95 *μ*g/mL, TPGS micelle 1.36 *μ*g/mL	[122]

TPGS-emulsified PLGA-mPEG nanoparticles	PTX	IGROV1 ovarian cancer cells *in vitro* and *in vivo *	Decreased toxicity	N/A	[123]

TPGS-emulsified polymeric nanoparticles (TENPs)	PTX	A549 lung cancer xenograft models	Inhibition of tumor growth	N/A	[124]

Dendrimer-TPGS micelles	PTX, DTX	MCF-7 and A549 cancer cells	Enhanced solubility and increased cytotoxicity	N/A	[125]

TPGS-PLA micelles	Crizotinib, Palbociclib, and Sildenafil	A549 lung cancer cells	Improved cytotoxic effect	IC_50_ (*μ*M): Crizotinib 26.07, Palbociclib, 17.65, double treatment 17.47, and triple treatment 13.23	[126]

*α*-TOS-TPGS-2k nanoparticles	DOX	MCF-7 cells, CT26-tumor-bearing mice	Reduced cell growth, delay in tumor growth	*In vitro* IC_50_ (*μ*M): TPGS 22 ± 2, TPGS + TOS 10.1 ± 0.8, TPGS + TOS + DOX 1.4 ± 0.4	[128]

Nanoparticle	MH	A549 *in vitro* and *in vivo *	Enhanced toxicity	*In vitro* IC_50_ (*μ*M): free MH 56.23, TPGS-MH nanoparticles 28.89	[129]

Methoxy poly (ethylene glycol)-b-polycaprolactone (mPEG-PCL), Doxorubicin (DOX), Docetaxel (DTX), paclitaxel (PTX), poly(lactic-co-glycolic acid) (PLGA), poly(lactic acid) (PLA), and Morin Hydrate (MH).
